# Neural correlates of derived relational responding on tests of stimulus equivalence

**DOI:** 10.1186/1744-9081-4-6

**Published:** 2008-02-01

**Authors:** Michael W Schlund, Michael F Cataldo, Rudolf Hoehn-Saric

**Affiliations:** 1 Department of Behavioral Psychology, Kennedy Krieger Institute, Baltimore MD, USA; 2 Department of Psychiatry and Behavioral Sciences, Johns Hopkins University School of Medicine, Baltimore MD, USA

## Abstract

**Background:**

An essential component of cognition and language involves the formation of new conditional relations between stimuli based upon prior experiences. Results of investigations on transitive inference (TI) highlight a prominent role for the medial temporal lobe in maintaining associative relations among sequentially arranged stimuli (A > B > C > D > E). In this investigation, medial temporal lobe activity was assessed while subjects completed "Stimulus Equivalence" (SE) tests that required deriving conditional relations among stimuli within a class (A ≡ B ≡ C).

**Methods:**

Stimuli consisted of six consonant-vowel-consonant triads divided into two classes (A1, B1, C1; A2, B2, C2). A simultaneous matching-to-sample task and differential reinforcement were employed during pretraining to establish the conditional relations A1:B1 and B1:C1 in class 1 and A2:B2 and B2:C2 in class 2. During functional neuroimaging, recombined stimulus pairs were presented and subjects judged (yes/no) whether stimuli were related. SE tests involved presenting three different types of within-class pairs: Symmetrical (B1 A1; C1 B1; B2 A2; C2 B2), and Transitive (A1 C1; A2 C2) and Equivalence (C1 A1; C2 A2) relations separated by a nodal stimulus. Cross-class 'Foils' consisting of unrelated stimuli (e.g., A1 C2) were also presented.

**Results:**

Relative to cross-class Foils, Transitive and Equivalence relations requiring inferential judgments elicited bilateral activation in the anterior hippocampus while Symmetrical relations elicited activation in the parahippocampus. Relative to each derived relation, Foils generally elicited bilateral activation in the parahippocampus, as well as in frontal and parietal lobe regions.

**Conclusion:**

Activation observed in the hippocampus to nodal-dependent derived conditional relations (Transitive and Equivalence relations) highlights its involvement in maintaining relational structure and flexible memory expression among stimuli within a class (A ≡ B ≡ C).

## Background

Considerable evidence highlights a role for the hippocampus in mediating our ability to derive relations among stimuli [1-6] and maintaining representational flexibility [7]. These two skills underlie many types of complex performances and successful functioning of humans, and have previously been studied with serial transitive inference (TI) paradigms. While this type of *derived relational responding *[8] is ubiquitous in everyday life, it is also the focal point of many Stimulus Equivalence (SE) based clinical-educational interventions, which are designed to teach children and individuals with cognitive dysfunction conditional relations among dissimilar stimuli, such as words, pictures and objects. For example, during SE training an individual may learn that when presented the spoken word 'cat' (sample stimulus A1), selection of the printed word "CAT" (comparison B1), but not the printed word "DOG" (comparison B2) produces reward. This differential reinforcement procedure establishes the auditory-visual conditional relation A1:B1. With additional training, a second visual-tactile conditional relation may be established between the word "CAT" (sample B1) and the tactile properties of a real feline (comparison C1), relative to a canine (comparison C2), resulting in the conditional relation B1:C1. Decades of basic and clinical research has shown these trained 'premises' lay the foundation for the emergence of several new conditional relations that include Symmetry (B1:A1 and C1:B1), Transitivity (A1:C1), and Equivalence (C1:A1) ([9], but also see 8 for a different perspective). Thus, the resulting stimulus class (A1 ≡ B1 ≡ C1) contains elements that are conditionally related, but not hierarchically or sequentially related, which markedly differs from serially ordered stimuli employed in TI paradigms (e.g., A > B > C > D > E)

The present investigation coupled BOLD fMRI and a SE methodology to examine medial temporal lobe involvement in derived relational responding. Findings relating the involvement of SE in frontal-parietal and frontal-subcortical networks [10-12] would show consistency with other investigations using TI tests [2,6] and offer an additional investigative tool for understanding complex learning as well as the role of the hippocampus. However, medial temporal lobe involvement in SE has been elusive, but may be anticipated based on findings obtained using serial TI paradigms [3,6]. One potential reason prior investigations have not observed medial temporal lobe involvement particularly in the hippocampus, is that the baseline comparator conditions used also contained a formal relation, such as matching two identical circles [10] or an explicit rule [2]. Consequently, both experimental and baseline conditions may have elicited similar levels of hippocampal activation. In the present investigation, a comparator condition was designed that consisted of unrelated or unpaired stimuli [3]. These 'Foils' were constructed by recombining stimuli from different classes, such as A1:C2. The hypothesis that derived relational responding would be mediated by the hippocampus was assessed by contrasting activation elicited by each derived relation to activation elicited by Foils.

## Methods

Twenty healthy, right-handed adults participated. Subjects reported being between 18 and 50 years of age, right handed, free of medications affecting the central nervous system or the autonomic system for at least 2 weeks, and without a personal history of psychiatric disorder or a psychiatric history in first-degree relatives. Informed, written consent was obtained from all subjects according to the institutional guidelines established by the Johns Hopkins Human Subjects Protection Committee.

### Experimental conditions

#### Training

**Figure 1:**
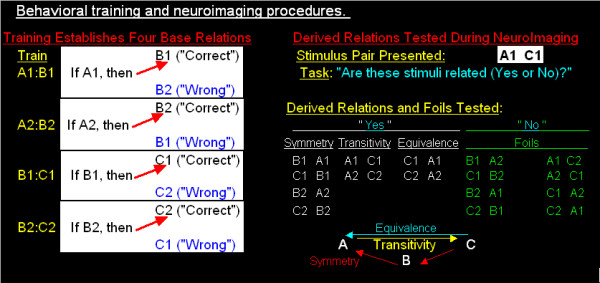
Behavioral training of premise pairs and SE testing during neuroimaging. The matching-to-sample pre-imaging procedure used to establish four premise pairs in two distinct classes (Class 1: A1:B1, B1:C1 and Class 2: A2:B2, B2:C2). During functional neuroimaging, two stimuli were presented (e.g., A2 C2) and subjects made yes/no judgments indicating whether the stimuli were conditionally related. Within-class derived relations consisted of Symmetry (B A; C B), Transitivity (A C) and Equivalence (C A) relations. Foils consisted of unrelated cross-class stimulus pairs that were not conditionally related (e.g., A1 B2).

Behavioral training occurred approximately three hours before neuroimaging and lasted one hour. Figure 1 shows the linear training structure (A-B-C) and the simultaneous matching to sample (MTS) procedure used to establish a set of class-specific 'premises'. On each trial, a sample stimulus was presented on the left side of a computer screen and two comparison stimuli presented on the right. Subjects were instructed that the sample stimulus was 'related' to one of the two comparisons and the task was to discover the relation by choosing one comparison (see [13,14] for additional discussion of MTS procedures). After comparison selection, feedback ('correct' or 'wrong') was provided. Two classes of stimuli were employed (designated 1 and 2), with each class containing three consonant-vowel-consonant triads, such as XUR. For simplicity, stimuli within each class will be referenced with a letter-number combination (Class 1 = A1, B1, C1; Class 2 = A2, B2, C2). Within each class, training established the conditional relations as follows: A1:B1, A2:B2, B1:C1 and B2:C2. Each conditional relation was trained individually in blocks of 20 trials until correct responding exceeded 90% accuracy, typically within 2–3 blocks. Finally, subjects completed SE tests in which a block of 20 trials contained the AB and BC premise pairs for each class intermixed with the following derived relations: Symmetry (B1 A1; C1 B1; B2 A2; C2 B2), Transitivity (A1 C1; A2 C2) and Equivalence (C 1 A1; C 2 A2). No corrective feedback was provided. For all subjects, response accuracy for each AB and BC premise and each derived relation exceeded 90% correct.

#### Neuroimaging

SE tests were completed during two BOLD functional neuroimaging runs. On each trial, a stimulus pair was presented (e.g., A2 C2). Instructions described the stimulus presentation, duration of trials, session length, and explained that the goal of the task was to press the 'yes' button if the stimulus pair were related and the 'no' button if they were not. As shown in Figure 1, SE tests to assess derived relational responding involved presenting Symmetry, Transitivity and Equivalence relations. Because subjects received exposure to each derived relation prior to imaging, subsequent activation patterns were not associated with acquisition, but rather with maintenance. Medial temporal lobe involvement in such derived relational responding was assessed by contrasting activation to derived relations relative to cross-class "Foils" constructed using stimuli from Class 1 and Class 2, such as A1 B2. Thus, the fundamental difference between derived relations and foils was the presence of an untrained conditional relation. A total of 36 Symmetry, 36 Transitivity, 36 Equivalence and 30 Foil trials were presented.

### Functional neuroimaging task and acquisition parameters

Subjects were placed in the scanner and handed a response box containing 'yes' and 'no' response buttons. Using an event related design, stimulus pairs were randomly presented for 2000 ms, followed by a blank screen averaging 3000 ms, which effectively 'jittered' stimulus presentations across time such that stimulus onsets were separated by an average of 5 s. Functional MRI images were obtained on a 3 T Philips MRI scanner while Eprime software controlled stimulus presentation rate and recorded timing and response data. Stimuli were presented on a rear screen monitor viewed through a mirror anchored to a standard head coil. After an initial series of sagittal T1-weighted localizers, a set of oblique T1-weighted images, angled parallel to the intercommissural line, were gathered. The fMRI data were acquired at the same slice locations. The T1 parameters were a repetition time (TR) of 500 ms and an estimation time (TE) of 11 ms. Functional MRI data were gathered using a single-shot echo planar imaging (EPI) sequence with a TR of 3000 ms, a TE of 50 ms, and a 90-degree flip angle. The matrix size was 64 × 64 and the field of view 24 cm, yielding voxels measuring 3 × 3 mm in plane. Using these parameters, 43 contiguous slices were obtained angled parallel to the intercommissural line.

### Functional neuroimaging analyses

For a subject's imaging data to be included in the analysis, head movement during the two functional runs was required to be limited to less than 2 mm. All preprocessing and data analyses were performed using statistical parametric mapping software, version 2 [15-18]. EPI images were slice-timing corrected to adjust for the lag between slices during each TR, corrected for head motion during scanning, and normalized to a standard template brain from the Montreal Neurological Institute (MNI) to get all participants into the same space. After normalization, voxels were resampled to 2^3 ^mm. EPI images were then spatially smoothed using a 6 mm full-width-half-maximum (FWHM) Gaussian kernel. High pass filtering was applied to the time series of EPI images to remove the low frequency drift in EPI signal and then subjected to a conventional two-level analysis. At the first level, individual-subject models were constructed in which a linear regression analysis was performed between the observed event related EPI signals and onset times of each derived relation (Symmetry, Transitivity, Equivalence) and the baseline condition (Foils) associated with correct responding. Subsequent contrast images were produced by performing voxel-wise comparisons between each derived relation and Foils. Contrast images were carried to a second 'random effects' level and subjected to ANOVA. The thresholds *P *< .005, uncorrected for multiple comparisons, and 20 contiguous voxels were employed. The location of voxels with significant activation was summarized by their local maxima separated by at least 8 mm, and by converting the maxima coordinates from MNI to Talairach coordinate space using linear transformations [19]. Coordinates were finally assigned neuroanatomic labels using the Talairach Daemon [20] and Talairach atlas [21].

## Results

### Behavioral

**Figure 2:**
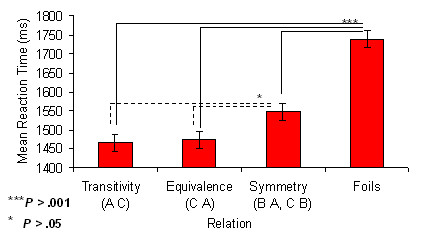
Mean reaction times associated with recognition of derived relations and foils. Response accuracy for each subject to each derived relation and Foils exceeded 90%. Reaction times were significantly faster to Transitive, Equivalence and Symmetrical relations relative to Foils (*P *< .001) and significantly faster to Transitive and Equivalence relations relative to Symmetrical relations (*P *< .05).

For each subject, response accuracy exceeded 90% correct for each derived relation and Foils during neuroimaging. Reaction times presented in Figure 2 reveal significantly faster responding to Transitive, Equivalence and Symmetrical relations relative to Foils (*P *< .001) and significantly faster responding to Transitive and Equivalence relations relative to Symmetrical relations (*P *< .05).

### Derived > Foils contrasts

**Figure 3:**
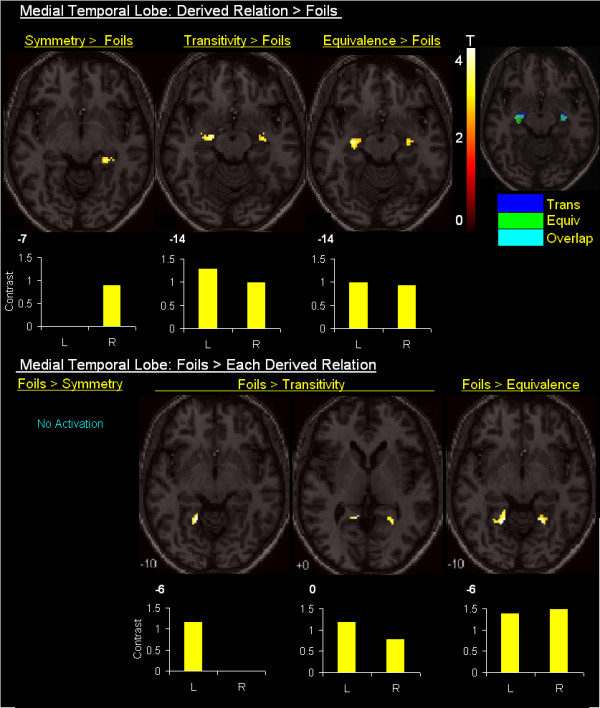
Derived < > Foils contrasts within the medial temporal lobe. The first row of statistical parametric maps highlights that Symmetry relations (B:A; C:B) contrasted with Foils (i.e, unrelated stimulus pairs, e.g. A1 C2) elicited activation within the right anterior parahippocampus, bordering the hippocampus, whereas Transitive (A:C) and Equivalence (C:A) relations contrasted with Foils elicited bilateral activation within a similar region of the anterior hippocampus. The second row of statistical parametric maps shows Foils contrasted Transitive (A:C) and Equivalence (C:A) relations elicited activation in the parahippocampus. Corresponding plots for each contrast highlight parameter estimate differences.

Table 1 highlights regions showing activation for each derived relation relative to Foils. Figure 3 shows that Transitive and Equivalence relations elicited bilateral activation in the anterior hippocampus, which overlapped considerably (see insert), while Symmetrical relations elicited activation in the nearby parahippocampus. These results suggest hippocampal involvement was limited to derived relations maintained by an intervening or nodal stimulus (i.e., B1 and B2).

**Table 1:** Regions differentially activated to derived relations relative to cross-class foils

			Talairach			
				
Contrast	Region		X	Y	Z	Volume (mm3)
Transitivity > Foils						
	Left	Posterior Cingulate	-4	-47	23	22
		Medial Frontal Gyrus	-12	61	12	469
		Medial Frontal Gyrus	-2	60	-6	(469)
		Hippocampus	-22	-14	-13	55
		Superior Temporal Gyrus	-53	-6	0	20
		Middle Frontal Gyrus	-32	41	-5	39
		Superior Temporal Gyrus	-40	17	-19	20
	Right	Anterior Cingulate	4	54	-1	(469)
		Superior Temporal Gyrus	48	-6	-6	69
		Inferior Frontal Gyrus	51	42	-11	30
		Medial Frontal Gyrus	14	51	7	22
		Hippocampus	34	-12	-13	69
Equivalence > Foils						
	Left	Hippocampus	-30	-16	-14	63
		Middle Frontal Gyrus	-30	42	-7	26
		Middle Temporal Gyrus	-38	-35	-3	29
		Caudate Tail	-32	-33	0	(29)
		Medial Frontal Gyrus	-8	59	14	47
		Medial Frontal Gyrus	-10	58	-6	54
	Right	Anterior Cingulate	2	27	-8	45
		Anterior Cingulate	4	5	-10	40
		Medial Frontal Gyrus	6	60	-5	(54)
		Hippocampus	30	-18	-11	20
Symmetry > Foils						
	Left	Paracentral Lobule	-6	-27	44	27
		Medial Frontal Gyrus	-16	43	14	29
	Right	Medial Frontal Gyrus	14	54	-6	64
		Anterior Cingulate	4	41	0	27
		Medial Frontal Gyrus	6	48	-6	(27)
		Parahippocampus	26	-35	-7	32
		Medial Frontal Gyrus	18	51	5	27
		Superior Frontal Gyrus	16	55	14	(27)

() Denotes secondary local maxima within cluster

### Foils > Derived contrasts

**Figure 4:**
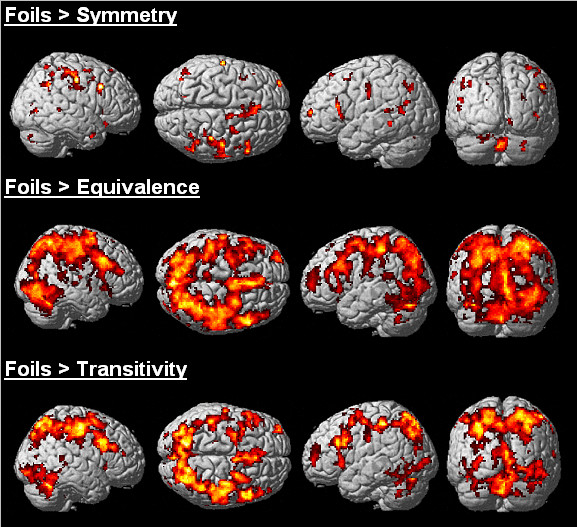
Differential activation to Foils relative to derived relations. Three-dimensional renderings of activation to Foils contrasted with Symmetrical, Transitive and Equivalence relations. Results highlight pronounced frontal and parietal activation to Foils relative to Transitive and Equivalence relations. These considerable magnitude differences appear to index conflict between recognition of incorrect stimulus relations relative to recognition of correct derived stimulus relations.

Relative to derived relations, Foils did not elicit activation in the hippocampus. Figure 3 shows Foils contrasted with Transitive and Equivalence relations elicited bilateral activation in the parahippocampus — no differences were observed when contrasted with Symmetrical relations. Table 2 and Figure 4 also highlights considerable activation to Foils in dorsal, inferior, and medial frontal regions, inferior and superior parietal regions, and middle and superior temporal regions, as well as in the thalamus, cerebellum, posterior cingulate and striatum. The differences in the extent of activation presented in Figure 4 appears to correlate with the reaction times differences presented in Figure 2. The considerable amount of activation observed to Foils relative to the derived relations, especially for 'nodal' relations, suggests discriminating the absence of a conditional relation may recruit a similar set of regions as discriminating the presence of a conditional relation, but to a significantly greater degree. This finding is not inconsistent with the idea that increased activation, particularly in frontal regions, reflects a conflict between an incorrect set of stimulus relations and a learned set of stimulus relations [22].

**Table 2:** Regions differentially activated to cross-class foils relative to derived relations

			Talairach			
				
Contrast	Region		X	Y	Z	Volume (mm 3)
Foils > Symmetry						
	Left	Anterior lobe	-12	-57	-22	122
		Inferior Frontal Gyrus	-46	24	15	140
		Middle Frontal Gyrus	-34	59	6	53
		Inferior Parietal Lobule	-48	-64	40	40
		Postcentral Gyrus	-59	-14	30	64
		Posterior Lobe	0	-71	-25	243
		Precuneus	-16	-60	42	30
		Middle Temporal Gyrus	-51	-61	25	(91)
		Superior Temporal Gyrus	-51	-54	14	91
	Right	Anterior lobe	2	-53	-9	97
		Inferior Frontal Gyrus	55	19	23	(155)
		Medial Frontal Gyrus	8	12	47	393
		Middle Frontal Gyrus	44	19	32	155
		Insula	38	24	10	42
		Middle Occipital Gyrus	42	-68	7	48
		Lingual Gyrus	16	-76	-3	113
		Inferior Parietal Lobule	51	-58	40	130
		Postcentral Gyrus	38	-27	46	440
		Posterior Lobe	4	-79	-20	(243)
		Precentral gyrus	44	-12	41	(440)
		Middle Temporal Gyrus	55	2	-29	32
		Superior Temporal Gyrus	48	-37	6	27
Foils > Transitivity						
	Left	Cingulate	-6	-10	39	52
		Inferior Frontal Gyrus	-30	22	6	(13448)
		Middle Frontal Gyrus	-38	54	1	222
		Lateral Globus Pallidus	-14	6	2	29
		Substania Nigra	-10	-16	-9	288
		Posterior Lobe	0	-69	-27	(10168)
		Lateral Posterior Nuc.	-16	-21	14	22
	Right	Cingulate	8	-7	45	55
		Medial Frontal Gyrus	14	-9	50	(55)
		Middle Frontal Gyrus	44	43	13	34
		Midbrain	4	-20	-14	(288)
		Middle Occipital Gyrus	30	-71	15	28
		Lingual Gyrus	16	-64	-5	10168
		Pons	10	-42	-33	34
		Posterior Lobe	38	-59	-19	(10168)
		Precentral Gyrus	38	-13	43	13448
		Putamen	24	2	9	20
		Middle Temporal Gyrus	48	-39	0	52
		Superior Temporal Gyrus	63	-42	9	30
		Ventral Lateral Nuc.	14	-13	10	(45)
		Pulvinar	16	-23	16	(45)
Foils > Equivalence						
	Left	Anterior lobe	-34	-51	-16	(42397)
		Caudate Body	-12	8	7	(460)
		Caudate Head	-12	15	-2	(460)
		Cingulate	-2	-33	33	178
		Middle Frontal Gyrus	-40	53	5	423
		Superior Frontal Gyrus	-30	55	17	(423)
		Lateral Globus Pallidus	-14	6	0	460
		Midbrain	-6	-24	-11	(1554)
		Paracentral Lobule	-16	-31	48	65
		Putamen	-30	-17	0	26
		Superior Temporal Gyrus	-61	-44	17	123
	Right	Claustrum	30	-3	17	72
		Inferior Frontal Gyrus	53	15	-6	48
		Insula	34	-23	16	(66)
		Midbrain	6	-24	-6	1554
		Inferior Occipital Gyrus	42	-76	-1	42397
		Inferior Parietal Lobule	57	-40	22	(129)
		Posterior Cingulate	6	-40	24	(178)
		Putamen	18	10	0	288
		Middle Temporal Gyrus	57	-62	12	23
		Superior Temporal Gyrus	44	-25	5	268
		Transverse Temporal Gyrus	53	-15	10	(268)

() Denotes secondary local maxima within cluster

## Discussion

The present findings highlighting activation in the hippocampus to nodal-dependent derived conditional relations (Transitive and Equivalence relations) and activation in the parahippocampus to cross-class Foils is generally consistent with results obtained using serial TI paradigms [3,6]. Accordingly, the present findings offer additional support for human hippocampal involvement in maintaining relational structure and flexible memory expression [7].

In the serial TI paradigm subjects learn a sequence of overlapping premise pairs (i.e., A > B > C > D > E) and inference (B > D) rests on knowledge of stimulus order. Commonly there is one, sometimes two, tests of inference. While prior investigations have shown hippocampal activation during inference, it remains unclear whether such findings are restricted to conditions involving serial learning. One argument offered against the serial TI paradigm as a test of inference is based on the grounds that it is an associative task with stimuli not falling along a linear dimension and inferences may be a function of a value transfer between and among the S+ and S- stimuli [23]. The results obtained in the present investigation using the SE paradigm appear to make some headway in clarifying hippocampal involvement in maintaining relational structure and inference. First, it was reassuring to observe hippocampal activation during Transitive relations (A:C) which parallels results reported during TI tests (B > D). But in addition, we also observed hippocampal activation during Equivalence relations (C:A). This finding demonstrates that hippocampal involvement is not dependent upon serial order within TI tasks and also that involvement is independent of the linear A, B, C training we employed. It is informative that Symmetry relations (B:A, C:B) did not elicit hippocampal activation. This finding may clarify that hippocampal activation reported during TI tests does not occur more generally to presentations of novel relations, but rather, activation is restricted to relations with intervening nodal stimuli. Lastly, prior investigations employing the serial TI paradigm have shown hippocampal activation during acquisition [6,12], with one study highlighting deactivation after learning was completed [4]. In contrast, we ensured there was accurate relational responding after training and prior to imaging. Therefore, our findings highlighting hippocampal activation during neuroimaging suggests the region may play a role in maintenance. Whether this is restricted to our use of stimulus classes remains unclear. Nevertheless, given the hypothesis that the hippocampus maintains relational structure, it seems expected that the hippocampus would show involvement after initial acquisition.

The application of SE paradigms holds the promise of opening up many new avenues of research on the role of the hippocampal complex in maintaining relational structure, especially across different sensory modalities. In the Introduction, we provided an example of a clinically based SE intervention used to establish derived relations among visual, auditory and tactile stimuli. There are no barriers we see that would limit the inclusion of taste, texture or odor into a class. This cross-modal feature of the SE paradigm stands in marked contrast with contemporary applications of serial TI paradigms where either necessity or convention dictates the use of stimuli from the same sensory modality. It is also plausible to suggest that while maintaining relational structure the hippocampal complex may play a central role in assigning functional properties to stimuli that are conditionally related. If the hippocampal complex mediates relations among stimuli, then changes in the functional properties of one stimulus would be expected to propagate to other related stimuli via the relational network. Numerous behavioral studies employing extensions of the basic SE paradigm have successfully shown how the function of one stimulus in a class, e.g. A1, may be transferred to other stimuli in the class, such as B1 and C1 [24]. This process is known as "transfer of function" and illustrates how stimuli may acquire functional properties through the relational network without direct experience. Here is seems important to note that transfer of function occurs to stimuli that are physically dissimilar, consequently, transfer is not simply a matter of *stimulus generalization*, which depends upon stimuli sharing physical properties. Relatedly, numerous behavioral studies have also successfully shown how changing the function of one stimulus in a class can change the functional properties of other stimuli in the class [25]. This process is referred to as "transformation of function" (for a review on transfer and transformation see [26]). In sum, the results of the present investigation, and probable role of the hippocampal complex in transfer/transformation of stimulus function, underscore the broad functionality of SE based preparations. New applications of the SE methodology promises to extend neuroscience research on medial temporal lobe functioning and higher cognitive functioning, as well as provide new insights into the effectiveness of SE based clinical treatments.

## Conclusion

Activation observed in the hippocampus to nodal-dependent derived conditional relations (Transitive and Equivalence relations) highlights its involvement in maintaining relational structure and flexible memory expression among stimuli within a class (A ≡ B ≡ C).

## Competing interests

The author(s) declare that they have no competing interests.

## Authors' contributions

MS, RH and MC were responsible for the fMRI design. MS carried out the data acquisition, analyses and each author contributed to the formation of the manuscript.
